# Precise Placement of Metallic Nanowires on a Substrate by Localized Electric Fields and Inter-Nanowire Electrostatic Interaction

**DOI:** 10.3390/nano7100335

**Published:** 2017-10-19

**Authors:** U Hyeok Choi, Jaekyun Kim

**Affiliations:** 1 Department of Polymer Engineering, Pukyong National University, Busan 48547, Korea; uhyeok@pknu.ac.kr; 2 Department of Advanced Materials Engineering, Hanbat National University, Daejeon 34158, Korea; 3 Department of Photonics and Nanoelectronics, Hanyang University, Ansan, Kyunggi-do 15588, Korea

**Keywords:** nanowire, deterministic assembly, dielectrophoresis, nanodevices

## Abstract

Placing nanowires at the predetermined locations on a substrate represents one of the significant hurdles to be tackled for realization of heterogeneous nanowire systems. Here, we demonstrate spatially-controlled assembly of a single nanowire at the photolithographically recessed region at the electrode gap with high integration yield (~90%). Two popular routes, such as protruding electrode tips and recessed wells, for spatially-controlled nanowire alignment, are compared to investigate long-range dielectrophoretic nanowire attraction and short-range nanowire-nanowire electrostatic interaction for determining the final alignment of attracted nanowires. Furthermore, the post-assembly process has been developed and tested to make a robust electrical contact to the assembled nanowires, which removes any misaligned ones and connects the nanowires to the underlying electrodes of circuit.

## 1. Introduction

Despite the abundance of functional nanowires and the demonstration of the elemental devices based on them, effective device fabrication remains unresolved. The fabrication challenges, including precise registration of nanowires and effective metallization for robust electrical connections to the substrate or circuit, are significant fundamental issues for bottom-up synthesized nanomaterials from a manufacturing perspective. Heterogeneous integration of functional nanowires with a complementary metal oxide semiconductor (CMOS) circuitry allows the possibility for multifunctional chip manufacture in which bottom-up synthesis and top-down fabrication technology merge. In most applications, assembly of a single nanowire at a predefined position and orientation with submicron registration accuracy is desirable, and especially preferable is a process that facilitates post-metallization of both ends of the nanowires. 

Some techniques, such as Langmuir-Blodgett film [1], fluidic directed-alignment [2], blown bubble film [3], chemically-functionalized patterns [4], and the contact printing method [5,6,7], have been proposed to integrate the nanowire on the substrate in a controlled fashion while keeping pace with the advances in nanowire synthesis and characterization. Their abilities, however, are limited to the production of the ordered nanowire arrays with some degree of orientation. Contact printing on the e-beam patterned photoresist trench and subsequent liftoff delivers a possible method to organize a single nanowire array on the substrate [8], yet accurate alignment of a single nanowire to the desired location still appears practically impossible. 

Alternating current (AC) dielectrophoresis (DEP) utilizes the translational motion of polarizable neutral objects in the liquid medium under the inhomogeneous electric field [9]. Since most of the metallic and semiconducting nanomaterials are more polarizable relative to non-aqueous media, they are attracted to the electrode gap where the high electric field is induced across the gap. As a result, DEP manipulation has been used to fabricate nanodevices by bridging the nanowires and nanotubes across the electrode gap [10,11,12]. Recently, AC DEP alignment made remarkable progress in accomplishing the precise alignment of a single metal nanowire [13,14,15], a semiconducting nanowire [16], nanotube device array [17], and even biologically-different nanowires on a single chip by their sequential DEP assembly [18]. A capacitively-coupled electrode structure [13,14,17,19] has been widespread to fabricate a large number of devices in parallel where biasing the underlying electrode causes the discrete surface electrodes to acquire a similar potential. In such schemes, strong DEP force between the surface electrode tips solely attracts the nanomaterials and is also responsible for their final alignment. In our recent experiment of the dielectric-covered parallel electrode structure [15,18], it was observed that the nanowires assembled at the electrode gap are mobile along the gap and determine their final position relative to the adjacent nanowire, hinting that the nanowire electrostatic (ES) interaction can assist the DEP force to improve the yield for deterministic nanowire placement. Here, we present a generic and versatile deterministic bottom-up integration of nanowires, using a novel aspect of DEP nanowire attraction and nanowire-nanowire ES interaction, where a large number of a single nanowire alignment with excellent registration accuracy is readily attained. The post-assembly process to fabricate the individual nanoscale device array and its perspective associated with the nanowire assembly are also discussed.

## 2. Experiment Details

The narrow metal electrode tip structure (Ti/Au = 20 nm/50 nm), fabricated on a silicon substrate, consists of ~1 μm thermally-grown oxide. Electrode tips, 5 μm long and 1 μm wide, are intended to form a 4 μm electrode gap from which a higher electric field region is induced. In order to prevent shorting the electric field between the biased electrodes, a dielectric layer of polymethylglutarimide (PMGI) with a thickness of 300 nm covers the entire substrate. The fluidic cell was used to confine the nanowire solution in an isopropanol to demonstrate this spatially-controlled alignment. An alternative to producing the spatially-controlled dielectrophoretic force on a substrate is to form a recessed photoresist well at the gap between the biased electrodes. This process applies a dielectric layer of PMGI (0.9 μm thick) on the substrate by interdigitated metal electrode fabrication. Following the lithographical patterning of a 7 μm long and 2 μm wide rectangle well array at the electrode gap using an imaging photoresist of BPRS-100, the subsequent ultraviolet ozone exposure of PMGI layer forms a ~0.4 μm deep well. The BPRS-100 photoresist layer is washed from the substrate using an acetone and isopropanol spray. 

Rhodium (Rh) nanowires used in this study were synthesized by Rh in the pores of commercial anodized aluminum oxide (AAO) membranes (Anodisc25, Whatman Scientific Inc., Pittsburgh, PA, USA). The nanowires are released by selectively etching the membrane in 3.0 M NaOH with a sonication for 30 min. The synthesized nanowires had nominal diameters of 200 nm and lengths of ~7 μm, which were determined by the pore diameter of the template and the electrodeposition time, respectively. Then, the nanowires are stored in the isopropanol prior to the assembly.

After the nanowire suspension is introduced into the fluidic cell, a sinusoidal bias with *V*_pp_ = 10 V, peak-to-peak voltage at the fixed frequency (ω = 100 kHz) is applied between the electrodes, which creates an inhomogeneous electric field in the electrode gap. The electric field strength and the dielectrophoretic force distribution was predicted using COMSOL Multiphysics^®^ software.

For the characterization and inspection of nanowire assembly, the optical microscope (BX-61, Olympus Co., Tokyo, Japan) using the bright and dark-field modes and a field-emission scanning electron microscope (FESEM) (S-4800, Hitachi Co., Tokyo, Japan) were used. The electrical measurement of the Rh devices was carried out using the semiconductor parameter analyzer (4156C, Agilent Co., Santa Clara, CA, USA).

## 3. Results and Discussion

**Figure 1:**
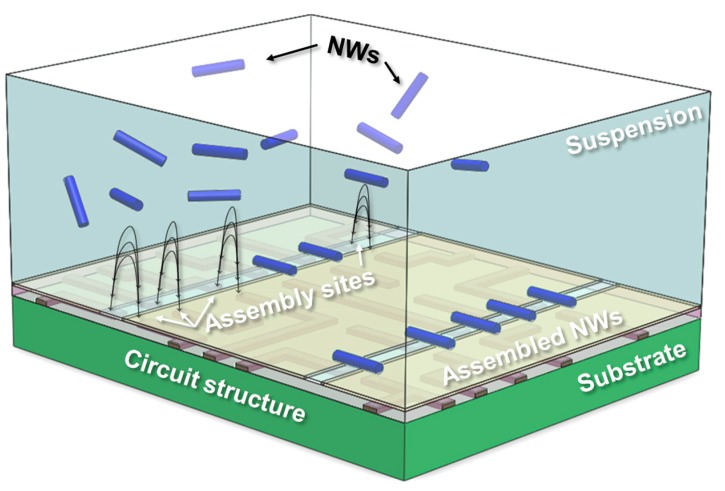
Illustration of deterministic nanowire assembly at the predetermined locations of the CMOS chip where the data acquisition, processing, and transmission can be fulfilled by multileveled CMOS circuitry. The functional nanowires suspended in the aqueous solution can be predominantly attracted and assembled to the desired positions relative to the underlying circuit while the wide metal electrode array effectively shields out any parasitic electric field. The post-assembly process will be followed to complete the individual device’s array connected to the circuit.

Figure 1 illustrates our scheme for achieving the deterministic nanowire assembly on a complex circuit structure. An array of wide electrode structures on a dielectric layer is designed to generate the inhomogeneous electric field from the gap while effectively eliminating the electric field by the capacitive coupling to the underlying circuit. For instance, a modern CMOS circuitry typically involves several alternating layers of complex metal leads and interdielectrics, which possibly cause unwanted sites to attract the nanowires by bias coupling. Figure 1 also depicts that the localized electric field from the assembly sites guides the nanowire movement and determines the final alignment, resulting in precise registration of nanowires relative to the features of underlying circuit. Furthermore, alignment defects, inherent to bottom-up assemblies, such as misaligned nanowires, can be relieved by the removal of the dielectric layer following the selective physical clamping of the nanowires at the assembly sites. The post-assembly processes, discussed at the end of this article, of the metal electrodeposition and the etching will complete the nanowire integration to the assembly sites and isolation between the devices. 

**Figure 2:**
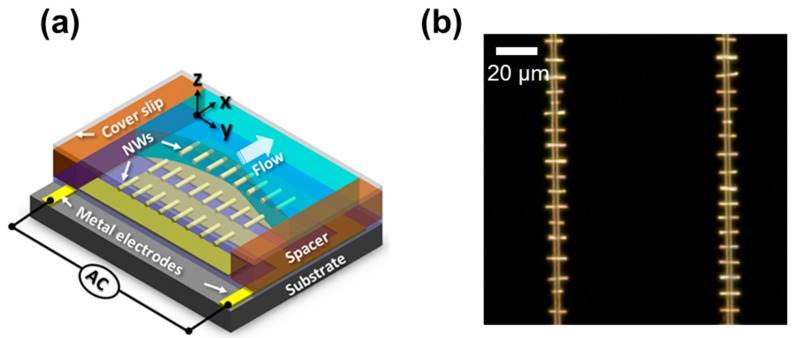
(**a**) Schematic of fluidic assembly cell used for the dielectrophoretic nanowire assembly. The flow in the figure indicates the direction of nanowire injection through the aperture of fluidic cell. The nanowire solution is confined under the cover slip during the nanowire alignment; and (**b**) the dark-field optical microscope image of uniformly-spaced 7 μm long Rh nanowire array assembled on the PMGI-covered electrode gap.

Figure 2a shows a schematic of the typical fluidic cell used in the nanowire assembly. The bottom substrate consists of the interdigitated metal electrodes, patterned by Ti/Au = 20 nm/60 nm evaporation, and the PMGI dielectric layer. The cover slip with an adhesive spacer is brought in contact with the substrate, forming a fluidic channel which is used to inject the nanowire solution. Constant evaporation through the aperture of the fluidic cell resulted in a uniform flow of solvent, which transports the nanowires into the substrate. Note that almost negligible flow velocity was found at the surface of the substrate. This low velocity is also beneficial in obtaining high alignment yield. A large quantity of nanowires with controlled length and diameter can be produced at relatively low cost via the electrodeposition using the AAO membranes. These nanowires can be assembled at the predefined locations on a substrate as shown in Figure 2b. One can notice that the nanowires are placed at the electrode gap while the meniscus of the nanowire solution passed. The specific position along the electrode gap can be precisely determined by the locally-enhanced electric field of the substrate, which will be discussed later in this article.

For the nanowires having a high-aspect ratio, the frequency-dependent dielectrophoretic force ***F****_DEP_* experienced by them in an AC electric field can be modified to [9,20]:

(1) $$F_{DEP}=\frac{\pi r^{2}\ell }{3}\epsilon_{1}R_{e}\left(K\right)\nabla E^{2}$$

**Figure 3:**
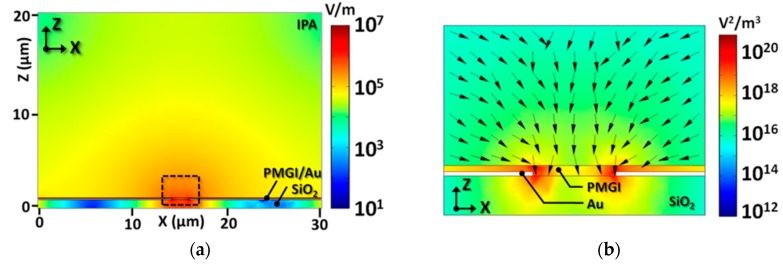

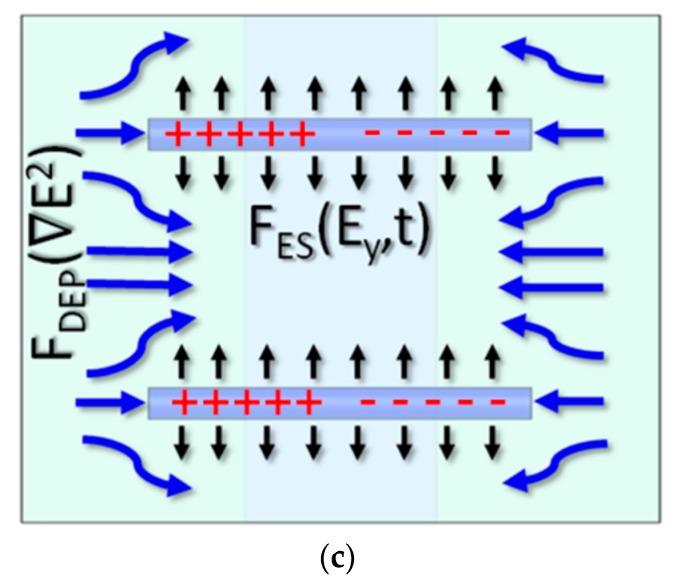
(**a**) Distribution of the electric field on *Z*–*X* plane and (**b**) the dielectrophoretic (∇***E***^2^) force simulation in the vicinity of electrode gap of the alignment structure based on the parallel electrode. The contour and normalized arrow map indicate the magnitude and direction of the DEP force, respectively. The DEP force is pointing toward the gap at all points, directing the nanowire deposition between the biased electrodes. (**c**) A schematic illustration of the DEP force (***F****_DEP_*, blue arrows) from the biased electrode and electrostatic interaction (***F****_ES_*, black arrows) between the polarized nanowires at the electrode gap. Crosses (+) and dashes (−) in red illustrate the induced charge of the nanowires by the biased electrodes. This combination of ***F****_DEP_* and ***F****_ES_* produces the ordered nanowire array with a uniform spacing from the suspension.

Where *r* is the radius of a nanowire, ℓ is the nanowire’s length, $\epsilon_{1}$ is the permittivity of a nanowire, $R_{e}\left(K\right)$ is the real part of the Clausius-Mossotti factor, and ∇***E***^2^ is the gradient of the square of the electric field strength. The Clausius-Mossotti factor accounts for the screened polarizability of the nanowires along the longitudinal direction in a liquid medium. Figure 3a shows that biasing to the metal electrodes creates the electric field directing toward the nanowire solution and, simultaneously, a long-range dielectrophoretic force [***F****_DEP_*(∇***E***^2^)] that attracts and orients the polarized nanowires to span the narrow electrode gap. The zoomed-in view of dielectrophoretic force in Figure 3b indicates the direction of polarized nanowires in the solution. It can be suggested that the nanowires tend to be attracted toward the electrode gap while biasing to the underlying electrodes.

In addition to the dielectrophoretic nanowire attraction, it is also important to recognize that the aligned nanowires maintain uniform spacing between them. This phenomenon can be attributed to the electrostatic (ES) interaction between the polarized nanowires. Specifically, the assembled nanowires are mobile along the electrode gap and tend to repel each other because their induced dipole moments are parallel to each other, making it possible for them to be redistributed by an adjacent nanowire. Since the polarization at the surface of the nanowires results in the Coulombic force between them along the y-direction, we can simply estimate ES force (***F****_ES_*) as follows:

(2) $$F_{ES}=\frac{Q^{2}}{4\pi \epsilon \;r}=QE_{y}$$

where *Q* is the induced charge on the half of the polarized nanowire, ϵ is the permittivity of solution, *r* is a distance between the nanowires, and ***E****_y_* is the magnitude of the y-component of the electric field. It can be approximated that ***F****_ES_* is proportional to ***E****_y_* because of the same polarization (*Q*) of the batch-fabricated nanowires in Equation (2). Using the electric field distribution, one can visualize the repulsive ES interaction between the assembled nanowires. Figure 3c shows the y-component of the electric field (***E****_y_*) from two adjacent nanowires, and ***E****_y_* is responsible for the repulsive force between them. This repulsive force plays an important role in achieving the high-yield deterministic assembly on the patterned substrate, which will be discussed in a later section. The metallic nanowires in the array tend to screen the electric field at the electrode gap. However, the electric field is still present between the assembled nanowires, as depicted in Figure 3c. Therefore, it is likely that the nanowires would be attracted in between them, consequently creating the dense array.

Producing a uniformly-spaced nanowire array appears to be a significant advance in the heterogeneous nanowire integration scheme. As stated earlier, it is often more desirable, however, to fabricate a large number of single nanowires at the predetermined locations in parallel. Precise nanowire registration with submicron accuracy for integration with modern CMOS chip is also required. To meet these goals, it is necessary to refine DEP nanowire assembly based on understanding the electromagnetics of this assembly system. Our earlier work [15,18] demonstrated that the localized electric field from the recessed lithographically-patterned region preferably attracted and aligned the nanowires from the solution. The finger-like electrode design [14,16,17,19] has been also a popular technique to assemble the single nanomaterials between a pair of electrode arrays where the localized electric field arises. Here, we modified our interdigitated electrode structure in a way that the electric field intensity is spatially-controlled at specific positions along the electrode gap by adding a protruding electrode or lithographically-defined recessed area, resulting in preferential assembly of individual nanowires within assembly sites. 

**Figure 4:**
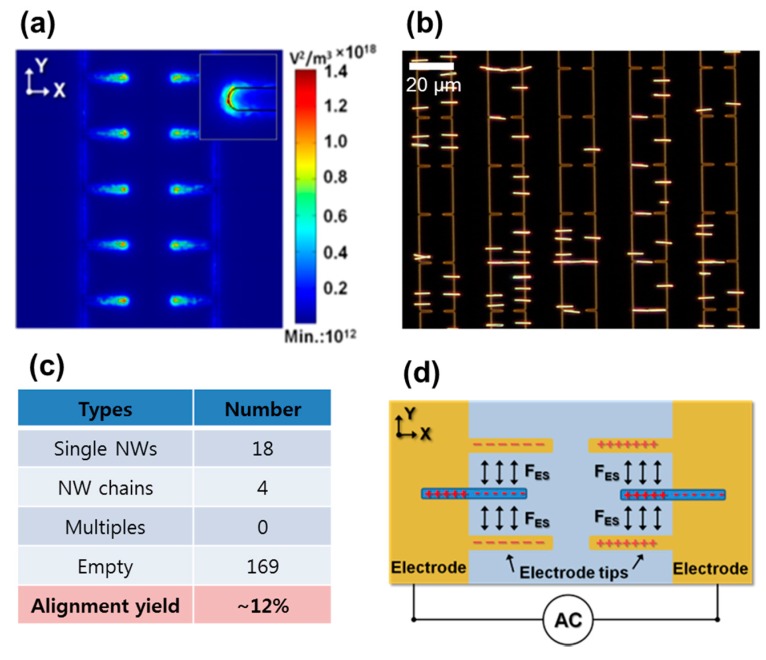
(**a**) Dielectrophoretic (∇***E***^2^) force simulation and (**b**) a dark-field optical microscope image of nanowire assembly on a PMGI-covered protruding electrode tip array. (**c**) A summary of nanowire assembly results of 192 sites after optical inspection; and (**d**) a schematic illustration of the repulsive electrostatic interaction between the assembled nanowires (blue color) and protruding electrodes (yellow color).

We performed the dielectrophoretic force simulation to understand the distribution of the alignment force, predict the nanowire assembly, and further improve the alignment yield and accuracy. This spatially-controlled dielectrophoretic force using an electrode tip is apparent from Figure 4a, exhibiting a relatively higher ∇***E***^2^ value on a substrate compared to elsewhere on the substrate. This simulation result indicates that the strongest dielectrophoretic force is induced at the electrode tips, which will favor the polarized nanowires. Since the electrostatic repulsive interaction between the polarized nanowires remains valid, expectedly, a predetermined, single nanowire device array between the electrode tips can be obtained. Figure 4b shows a dark-field OM image of Rh nanowire assembly over an electrode tip array on a substrate. In contrast to stronger dielectrophoretic force formation between the electrode tips; however, Figure 4b shows that most of the nanowires assemble at either of the edges of biased parallel electrodes, while only a small fraction of assembled nanowires appear at the gap of the protruding electrode tips. Visual inspection of a large area over a substrate was summarized in Figure 4c. Single nanowire alignment yield was as low as ~12%. This discrepancy between the theoretical expectation and experimental results appears counterintuitive. Considering another perspective of nanowire attraction and alignment at the spatially-controlled electric field locations could narrow the explanation of this discrepancy. That is to say, the relative magnitude of dielectrophoretic force for the spatially-controlled nanowire alignment could be one of the factors affecting alignment yield.

Taking the electrostatic force interaction between the assembled nanowires and protruding electrode tips into account is worthwhile. As illustrated in Figure 4d, consideration of the polarities of the biased electrodes and assembled nanowires reveals that the protruding electrode tip and outer half of the assembled nanowires are under the same phase of AC bias. As a result, the nanowires assembled at the edges of biased electrodes experience the repulsive force from the protruding electrode tips, keeping the assembled nanowires some distance from those electrode tips. Using a narrow, protruding electrode tip array structure, therefore, converted the ordered nanowire array, deterministically, led to unfavorable alignment on the electrode edges due to the repulsive electrostatic interaction between the polarized nanowires and oppositely-polarized electrode tips.

**Figure 5:**
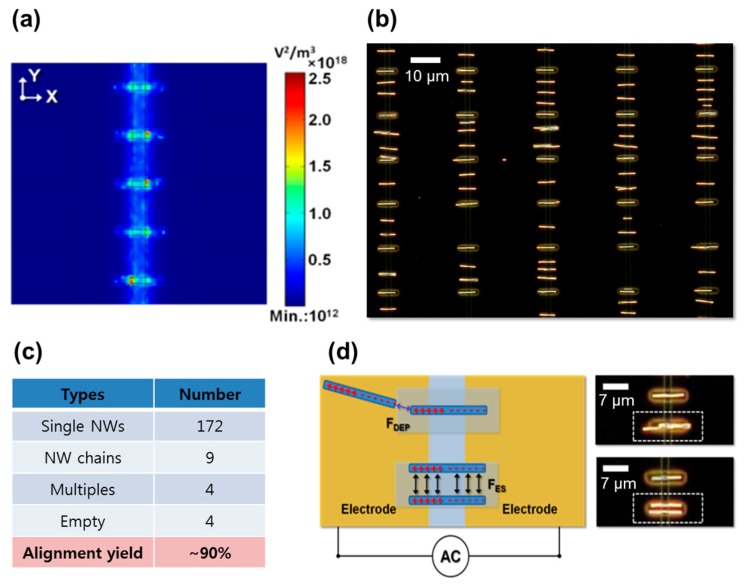
(**a**) Dielectrophoretic (∇***E***^2^) force simulation and (**b**) a dark-field optical microscope image of nanowire assembly on the recessed well array. (**c**) The summary of nanowire assembly results of 192 sites after optical inspection; and (**d**) a schematic illustration of attractive and repulsive electrostatic interactions between the assembled nanowires and incoming nanowires for chain formation and multiple nanowires in the well.

An alternative to producing the spatially-controlled dielectrophoretic force on a substrate is to form a recessed photoresist well at the gap between biased electrodes. Figure 5a shows the dielectrophoretic force simulation at the cross-sections of the *X*–*Y* plane of the nanowire alignment structure with a recessed photoresist well. This simulation predicts that the dielectrophoretic force attracts the nanowires from the suspension toward the electrode gap and determines their final alignment within recessed wells. A representative image of an experimental nanowire assembly with this structure, as shown in Figure 5b, shows that the nanowires are preferentially assembled at the locations defined by the lithographically-recessed dielectric region. Not surprisingly, several nanowires are also found at the electrode gap between the assembly sites, where a relatively low dielectrophoretic force also exists. Importantly, the assembled nanowires at the electrode gap, not within the recessed well, still maintain the uniform spacing between them and also span the electrode gap symmetrically, which coincides with the discussion for uniformly-spaced nanowire array formation. This result suggests that the electrostatic repulsive interaction between the assembled nanowires remains even after the nanowires’ alignment. Based on the low-magnification optical microscopic image over a large area (not shown here), statistical analysis of the assembly yield and related defects are summarized in Figure 5c. The yield of a single nanowire assembly, counted as a success, is a single nanowire trapped in a photoresist well. The yield is as high as ~90% and is reproducible and reliable. A small fraction (~5%) of the chained and multiple nanowires, considered assembly defects, are also found among the assembly locations.

Contrary to the protruding electrode tip structure, the recessed photoresist well favors the alignment of polarized nanowires in terms of the dielectrophoretic and electrostatic forces. The nanowires, assembling at the electrode gap, outside the desired, predetermined locations, can be attracted to the stronger dielectrophoretic region, namely that of the recessed photoresist well. Since the electrostatic repulsive interaction between the assembled nanowires remains viable, the nanowires’ alignment between the recessed wells can also assist positioning nanowires at the recessed well region through redistribution. Consequently, the predetermined assembly can be accomplished not only by direct nanowire alignment at the recessed well region, but also by the redistribution of aligned nanowires due to the repulsive force between them. 

Figure 5d shows the representative schematics of an electrostatic interaction between the assembled nanowires and relevant optical microscopic images of 7 μm long Rh nanowires assembled within a recessed photoresist well array. The assembled nanowires in the well still remain polarized as long as the AC bias is biased to the underlying electrodes. These polarized nanowires affect the incoming nanowires by virtue of the electrostatic attraction. Thus, one can easily find both the end-to-end nanowire chain formation (upper and right image of Figure 5d) and multiple nanowires in the well with a repulsion (bottom and right image of Figure 5d). Chained nanowires can be attributed to the mutual dielectrophoresis between polarized nanowires. As a result, this dielectrophoretic force, with a moderate strength, could attract a polarized nanowire toward a photoresist well already occupied by a nanowire. Notably, the electrostatic repulsive interaction makes the assembled nanowires within a well repel each other and, thus, pushes against the walls of the recessed photoresist well. The nanowire bundles relate to defects of nanowire batch-synthesis in which nanowires physically join during electrodeposition. In order to minimize these defects, the repetitive injection of low-concentration of nanowire solution is desired. 

The ability to assemble a single nanowire at a precise position on a substrate with a high yield and submicron registration accuracy leads the way for reliable, reproducible fabrication of electronic devices on substrates or as circuit structures. Since the assembled nanowires need to be electrically connected to the underlying electrode, the relevant post-assembly process requires development. In previously-reported assembly methods of exposed electrode structures [13,17,19], electrical contact between the nanomaterial and the metal electrode, apparently, occurs at the moment of alignment. As a result, the electrical contacts are not readily controlled, leading to variation in contact resistance, which eventually requires post-metallization [13], and even thermal annealing to lower the resistance [17]. Due to the configuration of the cylindrical-shaped devices and the flat electrode, metal evaporation or sputtering only covers a half or two-thirds of the assembled nanowires, possibly implying another source of nonuniformity in large numbers of devices. Making a reproducible and low contact to the nanowires provides a new challenge in fabricating electronic device arrays from a manufacturing perspective.

**Figure 6:**
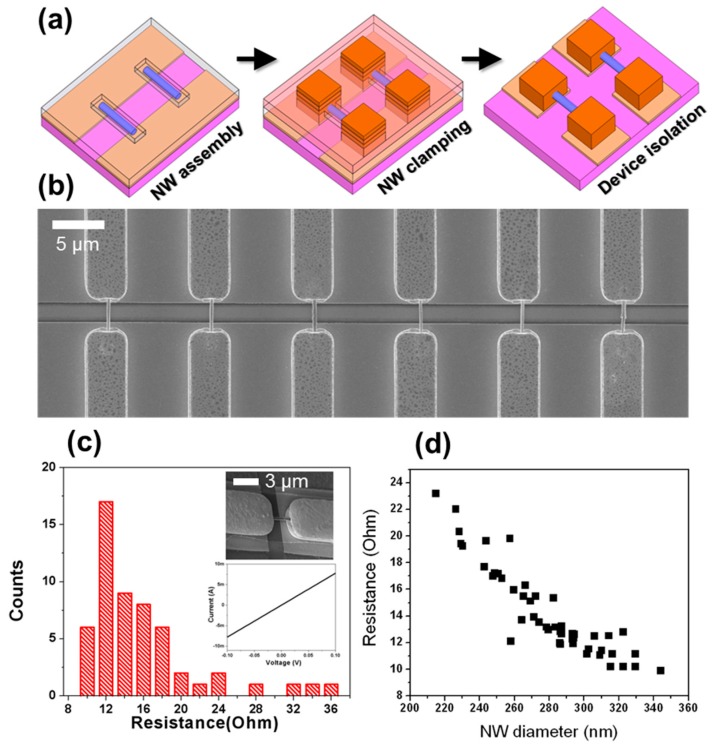
The post-assembly process for individual device fabrication. (**a**) An illustration of the processing steps for completing the single Rh nanowire device array. Nanowire assembly at the photoresist well, the electrodeposition for metal contact, and the dry etching for device isolation are presented sequentially. Purple: thermally-grown silicon oxide, yellow: the metal electrode for biasing, light blue: PMGI dielectric layer, light red: BPRS-100 photoresist, orange: electrodeposited gold; and blue: nanowires; (**b**) Scanning electron microscope image of a single Rh nanowire array clamped by the gold electrodeposition where the misaligned nanowires are released by PMGI removal; (**c**) The histogram for the resistance distribution from ~50 single Rh nanowire devices contacted by the wrap-around gold electrode. Representative current-voltage curve of single Rh nanowire device is placed as an inset showing the ohmic contact. The zoomed-in FESEM image exhibits the measured device consisting of the suspended Rh nanowire and metal contact; (**d**) A plot the resistance of single Rh nanowire vs. the nanowire diameter, suggesting that the variation in the Rh nanowire diameter is a major source that affects its resistance.

The functional nanowires, assembled in the recessed photoresist wells need to be connected to the underlying metal electrodes for electrical contact. As illustrated in Figure 6a, the post-assembly process using electrodeposition and dry etching is the proposed method to be accomplished following nanowire assembly. The advantage of this post-assembly method is two-fold. First, this wrap-around type contact, mechanically sturdy and electrically low-resistant in nature, of the assembled nanowires is of great promise for integration and secure functionality of nanowires on a substrate or circuit. Since the electrodeposition process holds the assembled nanowires firmly without excessive damping, different types of nanodevices can be fabricated for a hybrid nanosystem via the same process steps. For instance, the nanoelectromechanical and nanoelectronic device arrays may populate a single chip. Secondly, the dimension of the contact via window for the electrodeposition is designed to clamp the assembled nanowires within a photoresist well or less than 1 μm away from it. It is very unlikely that nanowires would occupy a position within 1 μm range of an existing nanowire at the electrode gap because of the electrostatic repulsion between the assembled nanowires and through control of the concentration of the nanowire suspension. As shown in Figure 5b, the attracted nanowires are prone to be positioned between the assembly sites if filled with nanowires. As a result, the electrodeposition and subsequent PMGI removal process effectively remove misaligned nanowires from assembly sites. 

As proof-of-concept and control studies, Rh nanowires are used to demonstrate the versatility of this post-assembly process and characterize the resistance variation in the devices by contacting the electrodeposited metal electrodes. Figure 6b shows the scanning electron microscopic image of a single Rh nanowire array clamped by a 3 μm wide electrodeposited electrode. The misaligned nanowires between the assembly sites were removed following PMGI removal. Individual addresses of the clamped devices are possible by selective removal of biased electrodes in a way that electrically isolates the devices in an array. To measure the electrical resistances of assembled Rh nanowires, the electrical characterization of clamped single Rh nanowires occurred by probing the contact pads. A histogram for the resistances of the measured devices appears Figure 6c. The tilted scanning electron microscopic image in the inset of Figure 6c demonstrates the representative single Rh nanowire devices, clamped by gold electrodeposition and isolated by dry etching. The current-voltage (I-V) characteristics of ~50 single clamped devices were measured, and all of them were functional, exhibiting the reliability of the developed post-assembly process. The statistical distribution of resistances of completed Rh devices showed relatively broad variation. Its origin will be identified using electrical and electron microscopy analysis in the following paragraph.

The typical I-V of the measured device as shown in the inset of Figure 6c indicates that the electrical contact to the assembled nanowires is purely ohmic, as expected from the metal-metal contact. The single Rh nanowire devices exhibit a mean resistance of ~13.4 Ω with a standard deviation of ±3.4 Ω. The measured resistivity of Rh in this device configuration (10.45 × 10^−6^ Ω⋅cm) seems to be almost twice its bulk value (4.74 × 10^−6^ Ω⋅cm for bulk at 300 K), which can be attributed to the microstructure of electrodeposited Rh and its contact to the electrode [21,22]. To identify the source of the resistance variation, the scanning electron microscope images of the measured devices were thoroughly investigated and compared with their corresponding resistances. Measurement of nanowire diameter reveals that the diameters of Rh nanowires are well distributed, ranging from 200 nm to 350 nm, which is in positive agreement with the batch-synthesis of electrodeposited nanowires [23]. Note that high-magnification FESEM was used to estimate the diameter of assembled Rh nanowires. A plot of the nanowire diameter vs. the resistance in Figure 6d suggests that the diameter variation in the nanowires by the nanowire batch-synthesis predominantly accounts for device-to-device variation rather than arising from nanowire assembly and/or post-nanowire assembly processes. It also suggests that the wrap-around metal contact formation to the assembled nanowires appears irresponsible to the total resistance of devices. Accordingly, improving the device variation via a tight control of the dimensions of nanowires, i.e., top-down fabrication of nanostructures is highly possible.

## 4. Conclusions

In conclusion, the method described in this paper offers a promising approach to position the single nanoscale building block at the predefined locations, made possible by the combination of the dielectrophoretic force from the photoresist wells and the electrostatic force from the polarized nanowires. It was demonstrated that the post-assembly process via the electrodeposition and dielectric layer removal can effectively transfer the assembled nanowires, only trapped in the photoresist wells, to the metal electrode, producing a large array of mechanically- and electrically-robust nanodevices. This result can be considered a significant advancement toward the commercial viability of bottom-up nanodevice fabrication. In addition, we believe that this post-assembly process can readily combine functional nanowire devices, such as the chemical and biological sensors at the predetermined locations to the underlying logic circuit, highlighting the seamless integration of bottom-up functional nanowire assembly and the top-down fabricated CMOS circuit. A reasonably small device variation considering the distribution of the nanowire diameter further proves the potential and versatility of this approach for integrating a large number of identical devices, allowing us to study the signal-to-noise improvement by signal averaging theory. This approach can be also extended to other noble nanophotonic applications such as the plasmonics of metallic nanopattern and the nanoresonator devices. Furthermore, this assembly technique is not limited to the metal nanowire and can be applicable to the polymeric and the inorganic nanowires, even to the biological entities, which will eventually allow us to explore the arbitrary heterogeneous integration of nanomaterials on a rigid or flexible substrate.
